# Interannual variability in terrestrial dissolved organic matter advection to the eastern East Siberian Sea under contrasting Beaufort Gyre conditions

**DOI:** 10.1038/s41598-025-07732-w

**Published:** 2025-07-02

**Authors:** Mi Hae Jeon, Jinyoung Jung, Juyoung Son, Kyoung-Ho Cho, Eun Jin Yang

**Affiliations:** 1 Korea Polar Research Institute, 26 Songdomirae‑ro, Yeonsu‑gu, Incheon, 21990 Republic of Korea; 2 Division of Earth and Environmental System Science, Pukyong National University, 45 Yongso-ro, Nam-gu, Busan, 48513 Republic of Korea

**Keywords:** Biogeochemistry, Ocean sciences

## Abstract

Recent changes in Arctic Ocean circulation, particularly the weakening of the Beaufort Gyre (BG), have potentially significant implications for biogeochemical cycles. This study investigates how interannual BG variability influences the distribution of river water and terrestrial-derived dissolved organic matter (DOM) in the western Arctic Ocean by comparing observations from two contrasting years: 2019 (maximum BG strength: 0.48 × 10^−6^) and 2022 (minimum BG strength: 0.14 × 10^−6^). Under these contrasting BG conditions, we identified significant changes in both river water fraction (*f*_river_) and terrestrial-derived DOM (characterized by fluorescent DOM; FDOM) in the eastern East Siberian Sea surface waters. Specifically, the *f*_river_ increased by 37%, while high-molecular-weight terrestrial humic-like FDOM increased by 29% from 2019 to 2022. These increases coincided with enhanced cyclonic circulation over the Eurasian Basin and a weakened anticyclonic BG in 2022, facilitating the eastward expansion of Lena River-influenced surface waters. Our findings demonstrate that BG conditions play a crucial role in controlling the distribution of river water and terrestrial-derived DOM from the Eurasian rivers in the western Arctic Ocean. Continued BG weakening could allow terrestrial DOM to penetrate further into the BG region, potentially altering its biogeochemical cycles and carbon dynamics.

**Supplementary Information:**

The online version contains supplementary material available at 10.1038/s41598-025-07732-w.

## Introduction

The Arctic Ocean receives a disproportionately large amount of global river discharge, accounting for 11% of the total, despite comprising only 1% of global ocean volume^1^. This substantial freshwater influx delivers significant quantities of terrestrial material, impacting the biogeochemistry and ecosystem dynamics of the upper Arctic Ocean^2–5^. Large quantities of dissolved organic matter (DOM), including carbon, are sequestered within terrestrial permafrost^6,7^ and can subsequently be transported to the Arctic Ocean via river system^8–11^. Accelerated permafrost thaw driven by climate change is expected to increase the influx of terrestrial-derived DOM (tDOM) into the Arctic Ocean^12^. This tDOM influences coastal and shelf biogeochemical processes^13–15^, while also extending its impact across the broader Arctic Ocean^8,16,17^. Furthermore, the East Greenland Current exports significant terrigenous dissolved organic carbon (DOC) from Arctic rivers annually to the North Atlantic^16^, potentially significantly impacting global carbon cycles^12^. Once in the ocean, tDOM remains closely associated with river water^18^, although partial losses can occur through photodegradation, microbial degradation, and flocculation^19–22^. Despite these partial losses, tDOM is relatively resistant to complete degradation in the marine environment, making it an effective tracer for tracking river water distribution in the Arctic Ocean^17,18,23^.

The distribution of river water and tDOM in the upper Arctic Ocean is influenced by a complex interplay of factors, including surface ocean circulation, atmospheric forcing, sea ice dynamics, and river discharge patterns. Among these, surface ocean circulation—particularly the interactions between the anticyclonic Beaufort Gyre (BG) and the Transpolar Drift (TPD)—plays a critical role in modulating freshwater transport pathways^24^. The BG acts as a reservoir, accumulating freshwater and tDOM from rivers, while the TPD transports Eurasian river water and its associated DOM toward the central Arctic Ocean and Fram Strait. These circulation patterns are highly susceptible to atmospheric forcing^25–27^, with wind-driven motion in the Arctic alternating between anticyclonic and cyclonic regimes at 5–7 year intervals since the 1950s^28^. Since 1998, these regime shifts have been interpreted within the framework of the Arctic Oscillation (AO)^29^. A negative AO phase, characterized by a strong Arctic High over the Canada Basin, expands the BG and directs the TPD from the Laptev Sea over the Lomonosov Ridge, channeling Eurasian river runoff toward the Fram Strait. Conversely, a positive AO phase strengthens cyclonic circulation, shrinking the BG and shifting the TPD toward the Alpha-Mendeleev Ridge, thereby facilitating the eastward expansion of Eurasian river runoff^26^.

While the AO exerts a dominant influence, regional atmospheric patterns, such as the Beaufort High (BH), can modulate these large-scale effects^27^. Numerical simulations have shown that even during a positive AO phase, a negative BH perturbation can further weaken the BG and enhance the eastward spreading of Eurasian runoff^27^. This highlights the complex interplay between large-scale and regional atmospheric forcing in regulating western Arctic Ocean (WAO) circulation. A recent study further suggests that BH variability influences freshwater distribution in the BG, as Wang^30^ demonstrated a link between BH sea level pressure (SLP) and the spatial variations in freshwater. In addition, a weakening trend in the BG, potentially linked to shifts in BH, led to corresponding changes in water masses in the WAO^31–33^. For example, the shrinking of the BG has facilitated the eastward expansion of Atlantic-origin cold halocline waters, previously restricted to the Lomonosov Ridge, along the East Siberian Sea (ESS) continental margin^33^. This circulation change has triggered anomalous biogeochemical responses^31,32^, including enhanced surface phytoplankton blooms due to shoaling of the nutricline and the subsequent upward transport of Pacific-origin nutrients to the surface layer^32^. Additionally, model simulations indicate that since 2012, the BG has extended northward, resulting in an eastward shift of the TPD^34^. These observed and modeled changes in Arctic Ocean circulation patterns could substantially affect river water and tDOM distribution.

Observational studies have demonstrated that atmospheric circulation plays a key role in modulating the distribution of Eurasian river-influenced surface waters in the Laptev Sea and ESS^35–38^. In the ESS, alternations between anticyclonic and cyclonic circulation regimes control the position of the frontal zone between Pacific waters and local Arctic shelf waters^39^, thereby regulating freshwater transport. This atmospheric control extends to tDOM dynamics, as Pugach et al.^38^ showed that local atmospheric forcing influences tDOM distribution variability across the East Siberian Arctic Shelf (ESAS). Similarly, Hölemann et al.^37^ emphasized the combined effects of wind forcing and river discharge on tDOM distribution. However, most of these studies have been restricted to specific regions, particularly the Lena Delta and western ESS^35–37^, primarily investigating the influence of local wind fields within the ESAS on the distribution of river water and tDOM. In contrast, the eastern ESS (east of ∼160°E) remains one of the least studied regions regarding atmospheric forcing and its effects on tDOM distribution. This is likely due to the dominance of marine-derived DOM in this region, as Pacific inflow waters enhance phytoplankton production, thereby increasing marine-derived DOM contribution^40,41^. Jung et al.^5,42^ reported lower riverine DOC in the eastern ESS compared to the Chukchi Borderland. These findings suggest that, under historical conditions, tDOM inputs in the eastern ESS were relatively minor. However, the decline in Arctic sea ice has amplified the upper ocean’s response to wind forcing, increasing the BH variability^43^. This shift could alter the intrusion of Eurasian river water into the eastern ESS, leading to changes in the previously observed tDOM distribution. Nevertheless, the role of BG variability–particularly its recently observed weakening–in modulating the distribution of river water and tDOM in the eastern ESS remains poorly understood, with only a few model simulations providing initial insights into this process^27,34^.

While previous observational studies have examined the interaction between local atmospheric circulation within the ESAS and the tDOM distribution in the Laptev Sea and ESS^37,38^, this study builds upon those studies by investigating how BG variability influences the distribution of river water and tDOM in the WAO, with a particular focus on the eastern ESS. To quantify BG strength, we use the strength (BG_STR_), defined as the difference between maximum and minimum dynamic ocean topography (DOT) divided by the mean radius of the BG^44^. We compare hydrographic and atmospheric conditions during two cruises conducted in the summers of 2019 (strong BG) and 2022 (weak BG). We utilize fluorescent DOM (FDOM) measurements as a proxy for tDOM^45,46^, an approach supported by its strong correlation with lignin phenols^47^, unique biomarkers of tDOM^1,48^. Specifically, studies across the Arctic Ocean have shown that terrestrial humic-like FDOM effectively traces the transport of riverine DOC^5^. By examining the distribution of terrestrial humic-like FDOM during periods of contrasting BG conditions, this study provides new insights into how BG variability modulate the distribution and lateral advection of tDOM.

## Results

### Interannual variability of the BG

**Fig. 1:**
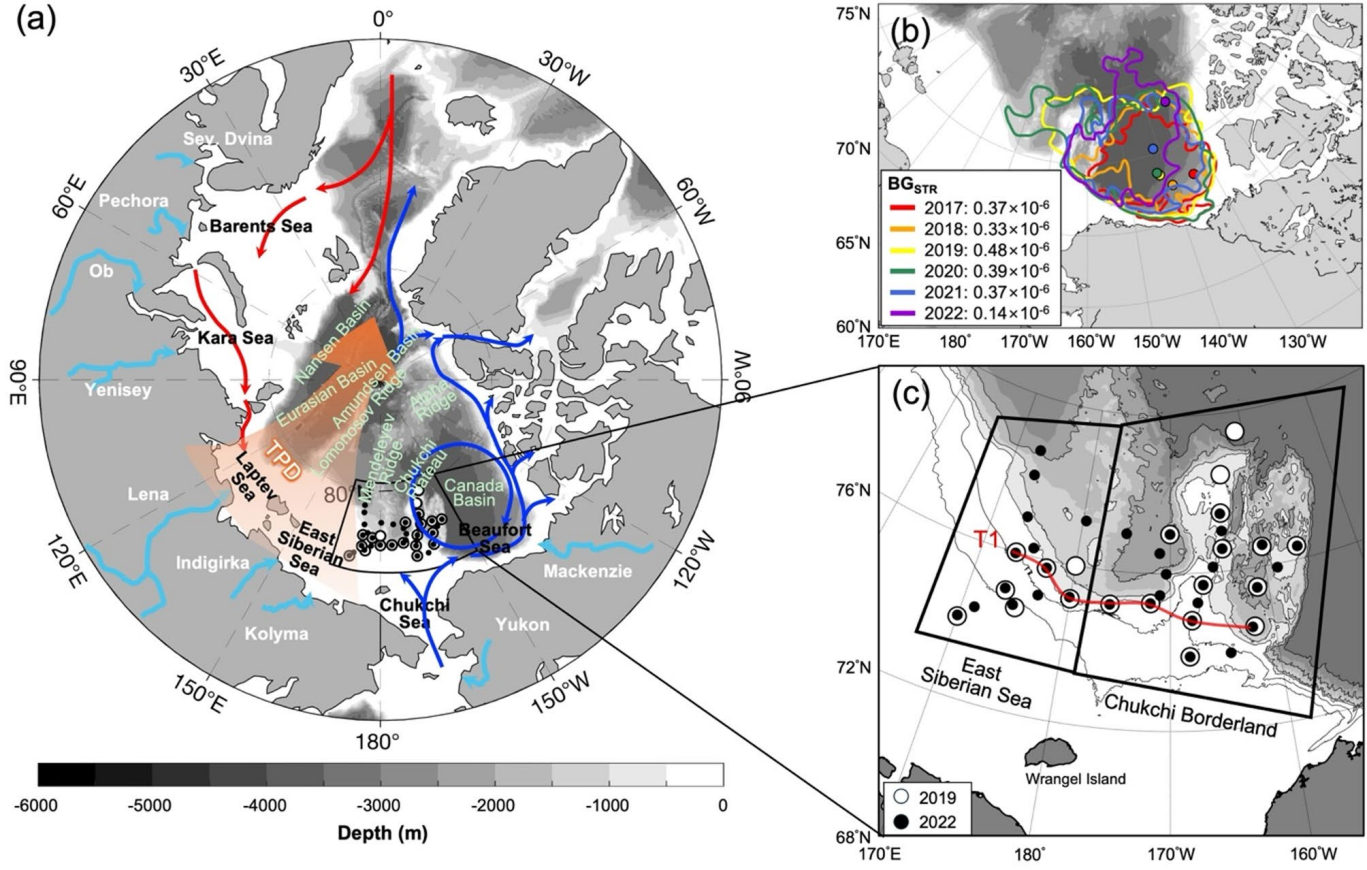
(**a**) Schematic of upper ocean circulation features. Blue and red arrows represent surface flows from the Pacific and Atlantic oceans, respectively. River discharge and transpolar drift (TPD) are indicated by light blue and broad orange arrows, respectively. The anticyclonic (clockwise) circulation over the Beaufort Sea represents the Beaufort Gyre (BG). The circulation features are based on the schematic from Charette et al.^4^. (**b**) The BG extent (BG_EXT_) (contours), the locations of the maximum dynamic ocean topography (DOT_max_) within the study region (dots inside the gyre), and the strength of the BG (BG_STR_) (as indicated in the legend) from 2017 to 2022. The BG_EXT_ is defined as the largest closed contour of the dynamic ocean topography (DOT), corresponding to the minimum DOT (DOT_min_) surrounding DOT_max_. The BG_STR_ is calculated as the difference between DOT_max_ and DOT_min_, divided by the mean radius of the BG. (**c**) Locations of sampling stations. White and black circles represent seawater sampling stations in the summers of 2019 and 2022, respectively. Geographic locations are divided into two regions: the East Siberian Sea and the Chukchi Borderland. The red line labeled “T1” refers to the hydrographic transect shown in Fig. 2. The Arctic maps in this figure were created using Python 3.9.19 (https://www.python.org/) with matplotlib 3.8.4 (https://matplotlib.org/) and basemap 1.4.1 (https://matplotlib.org/basemap/).

To investigate how changes in BG affect the distribution of river water and tDOM, we analyzed the BG_STR_ over a six-year period from 2017 to 2022 (Fig. 1b). We calculated BG_STR_ values^44^, which revealed substantial interannual variability ranging from 0.14 × 10^–6^ to 0.48 × 10^–6^. The analysis identified 2019 as having the strongest anticyclonic circulation (BG_STR_ = 0.48 × 10^–6^) and 2022 showing the weakest (BG_STR_ = 0.14 × 10^–6^). These two years represented the extremes in our dataset, with intermediate values of 0.37 × 10^–6^, 0.33 × 10^–6^, 0.39 × 10^–6^, and 0.37 × 10^–6^ observed in 2017, 2018, 2020, and 2021, respectively. Given the substantial variability in BG_STR_ (64% decrease from 2019 to 2022) and its potential to alter water mass distribution and transport pathways for freshwater and tDOM across the WAO, these two contrasting years were selected for detailed hydrographic and biogeochemical analysis.

### Hydrographic variability under contrasting BG conditions in 2019 and 2022

**Fig. 2:**
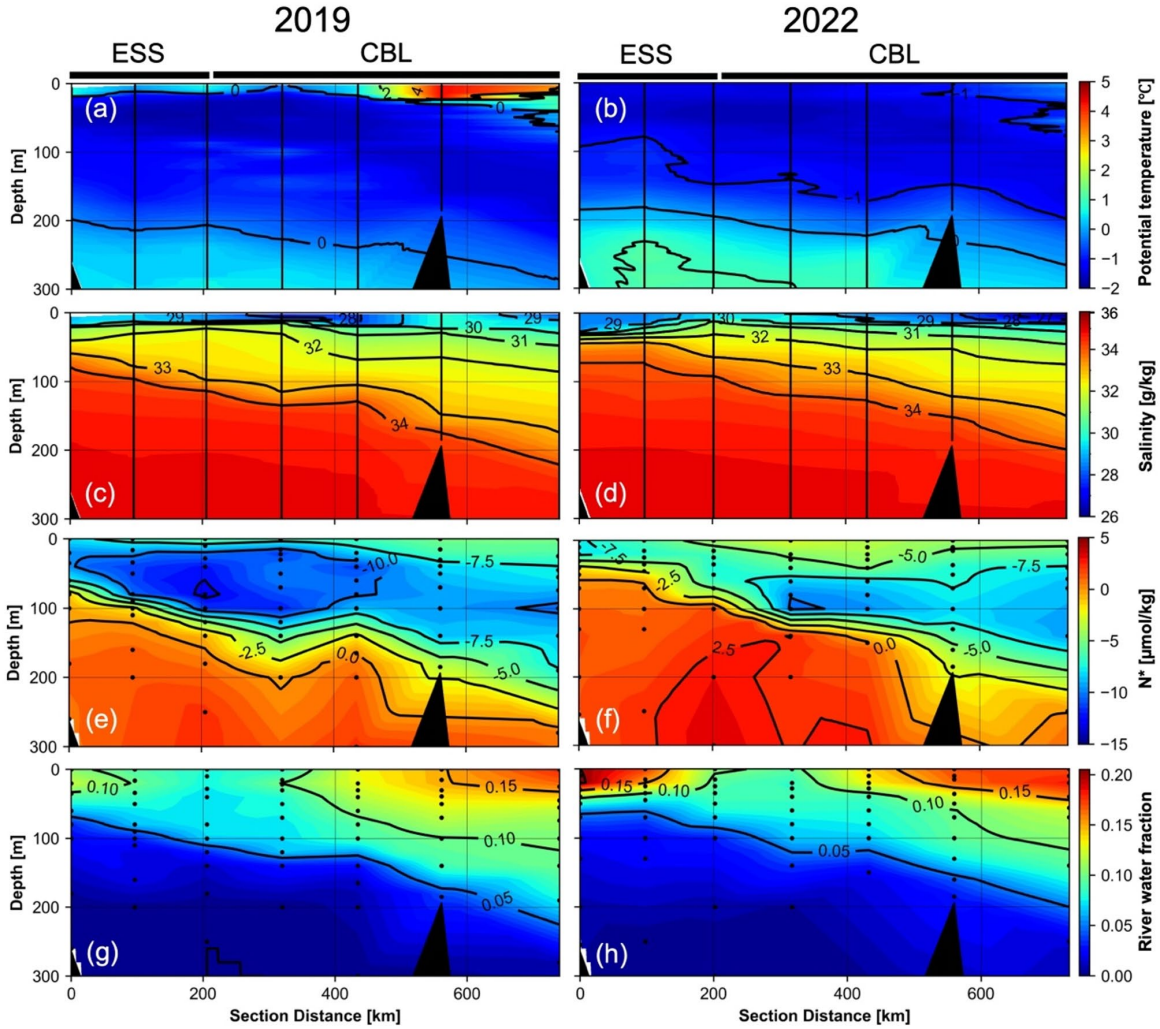
Vertical sections of (**a**,**b**) potential temperature (°C), (**c**,**d**) salinity (g/kg), (**e**,**f**) N^*^ (μmol/kg), and (**g**,**h**) river water fraction along T1 from the East Siberian Sea (ESS) to the Chukchi Borderland (CBL) (red line Fig. 1c) during the summers of 2019 (left panels) and 2022 (right panels).

Hydrographic measurements along T1 (Fig. 1c) revealed distinct differences in water mass structure between 2019 and 2022, corresponding to contrasting BG conditions (Fig. 2). Both years exhibited typical Arctic stratification, with a low-salinity surface layer overlying more saline Pacific- and Atlantic-origin waters. However, water mass distributions exhibited interannual variability.

The surface mixed layer (< 25 m) showed marked temporal variation in freshwater content. In 2019 (strong BG), low-salinity waters (< 29 g/kg) were confined to the Chukchi Borderland (CBL). In contrast, in 2022 (weak BG), these low-salinity waters were observed not only in the CBL but also in the ESS, suggesting enhanced freshwater input to the ESS. The upper halocline layer (50–200 m) was dominated by Pacific-origin water, which can be classified into Pacific summer water (absolute salinity (S_A_) = 31.15–32.15 g/kg, potential temperature (*θ*) maximum) and Pacific winter water (S_A_ ≈ 33.16 g/kg, *θ* minimum)^49^. The Pacific-origin halocline waters were characterized by cold temperatures (–1.00 ± 0.49 °C for 2019 and –1.08 ± 0.50 °C for 2022), moderate salinity (33.4 ± 1.07 g/kg for 2019 and 33.5 ± 0.99 g/kg for 2022), and a nitrogen deficit (N^*^ = –5.86 ± 4.70 μmol/kg for 2019 and –3.85 ± 4.90 μmol/kg). This upper halocline layer exhibited reduced westward penetration in 2022 compared to 2019. This is evident in the distribution of N^*^, which can be used as a tracer of Pacific-origin water transit^50,51^. In 2019, Pacific-origin water (marked by N^*^ < –5 μmol/kg, following Zhuang et al.^52^) extended into the ESS (Fig. 2e). However, in 2022, the –5 μmol/kg contour line in the ESS was positioned at shallower depths compared to 2019 (Fig. 2f), indicating reduced westward penetration. Simultaneously, Atlantic-origin lower halocline water (S_A_ ≈ 34.2 g/kg)^53^ expanded eastward, with its upper boundary in the ESS shoaling from 87–117 m in 2019 to 87–100 m in 2022 (Fig. 2c,d), implying an expansion of Atlantic-origin water into the eastern ESS in 2022 compared to 2019.

The distribution of river water fraction (*f*_river_) along T1 also exhibited notable differences between these two years (Fig. 2g,h). In 2019, *f*_river_ was primarily confined to the surface layers (< 50 m) of the CBL, with a maximum value of ~ 0.178 (Fig. 2g). In contrast, in 2022, *f*_river_ exhibited a broader and more pronounced distribution, with maximum values reaching ~ 0.205 observed in the ESS (Fig. 2h). Along T1, while *f*_river_ remained stable in the surface layers of the CBL between years (t-test, *p* = 0.47), it increased significantly in the surface layers of ESS (*p* < 0.05), from 0.070–0.120 (mean: 0.089 ± 0.015) in 2019 to 0.067–0.205 (mean: 0.128 ± 0.050) in 2022.

### Characterization of tDOM using fluorescence spectroscopy

**Fig. 3:**
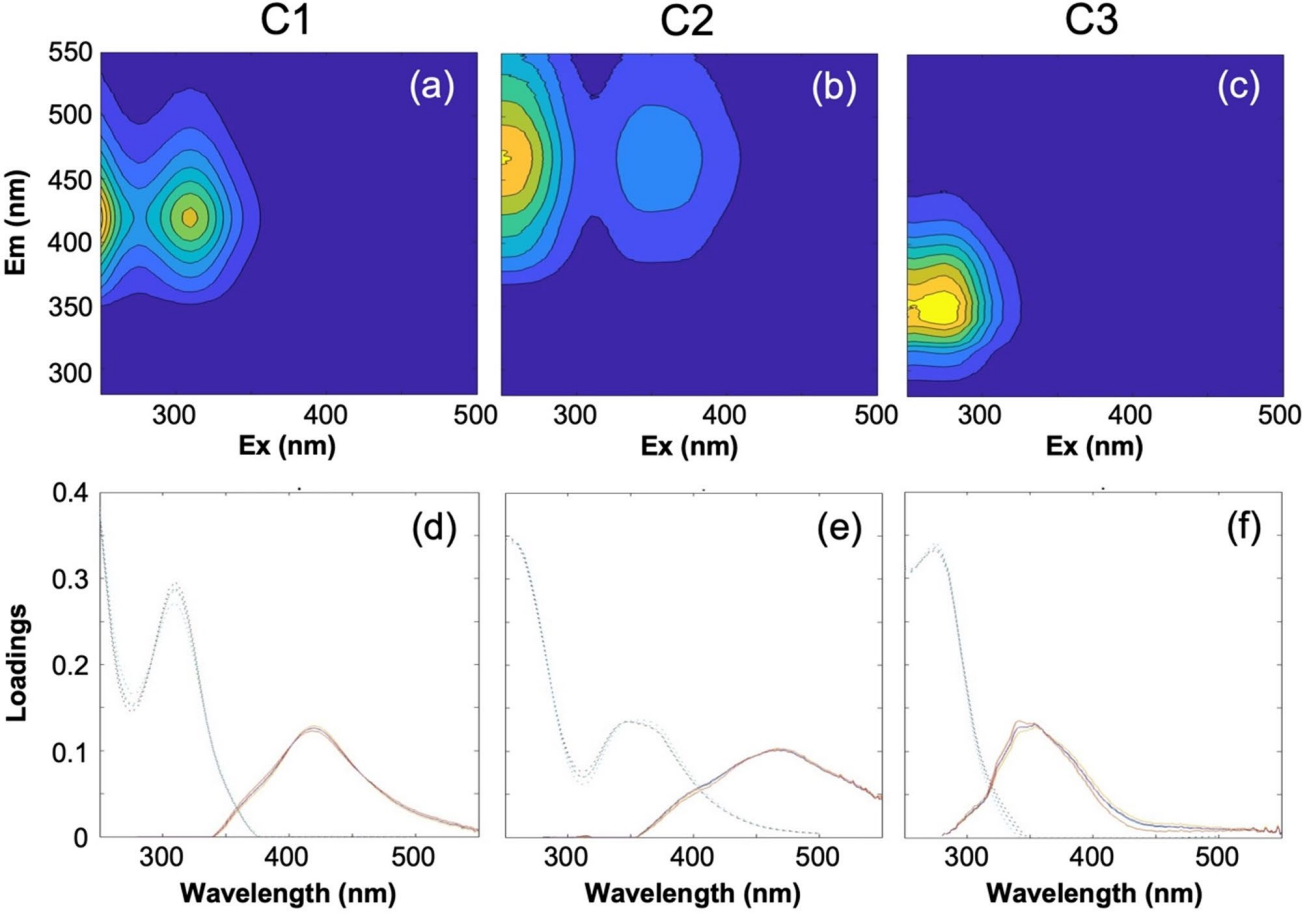
Three components identified by Parallel Factor Analysis (PARAFAC). (**a**–**c**) Fluorescence excitation–emission matrix contour plots. (**d**–**f**) Loadings of the three components, with the excitation (dashed lines) and emission (solid lines) spectra of three components.

To characterize the fluorescence properties of DOM and identify terrestrial components, we employed excitation–emission matrix fluorescence spectroscopy with parallel factor analysis (PARAFAC). A three-component model was established through rigorous validation using split-half analysis (Fig. 3). The fluorescent components were compared with those from other studies using the OpenFluor database^54^.

Component 1 (C1) exhibited maximum excitation (Ex) wavelengths at < 250 and 310 nm, with an emission (Em) wavelength at 419 nm (Fig. 3a,d). Based on its broad Em spectrum^55^, C1 was classified as humic-like FDOM. However, comparisons with the OpenFluor database revealed inconsistencies. Some matches showed that C1 aligned with terrestrial humic-like material^56–59^, whereas others suggested characteristics similar to marine humic-like fluorescence^60–62^. Due to this overlap, C1 was categorized as a mixture of terrestrial and marine humic-like components through visual inspection^63^. Component 2 (C2) emerged as our primary tracer for tDOM, exhibiting characteristic Ex peaks at < 250 and 350 nm and an Em peak at 468 nm. The spectral characteristics of C2 unequivocally identify it as a terrestrial humic-like fluorophore^64–68^, consistent with previous studies in the Arctic Ocean that have established its reliability as a tracer for tDOM^64,68^. Component 3 (C3, Ex/Em: < 250 (275)/353 nm) was assigned as tryptophan-like component^64,69,70^.

Given our focus on terrestrial inputs and their redistribution under varying BG conditions, subsequent analyses concentrated on C2. Its proven utility as a tDOM tracer in Arctic waters makes it particularly suitable for investigating tDOM transport patterns under the contrasting circulation regimes observed in 2019 and 2022.

### Spatial and temporal variability of river water and tDOM

The BG weakening between 2019 and 2022 led to changes in river water distribution, subsequently altering the tDOM distribution. Vertical profiles of *f*_river_ revealed region-specific responses to the changing circulation regime (Fig. S1). Over the 0–300 m depth range, *f*_river_ values varied from 0 to 0.199 (mean: 0.101 ± 0.052) in 2019 and from 0.002 to 0.183 (mean: 0.099 ± 0.052) in 2022 in the CBL (Fig. S1a), while in the ESS, values ranged from 0 to 0.128 (mean: 0.064 ± 0.038) in 2019 and from 0 to 0.214 (mean: 0.093 ± 0.067) in 2022 (Fig. S1b). The most pronounced change was observed within the 0–50 m layer in the ESS, where *f*_river_ increased from 0.095 ± 0.016 in 2019 to 0.141 ± 0.041 in 2022 (Fig. S1b).

This pattern was mirrored in the distribution of terrestrial humic-like C2. In the CBL (0–300 m), C2 intensities remained consistent, averaging 0.027 ± 0.006 R.U. (range: 0.014–0.039 R.U.) in 2019 and 0.026 ± 0.006 R.U. (range: 0.006–0.039 R.U.) in 2022 (Fig. S1c). In contrast, a substantial increase was observed in the ESS, where C2 increased from 0.029 ± 0.006 R.U. (range: 0.014–0.042 R.U.) in 2019 to 0.038 ± 0.018 R.U. (range: 0.010–0.092 R.U.) in 2022 (Fig. S1d). This variation was prominent within the 0–50 m layer in the ESS, where C2 increased from 0.029 ± 0.005 R.U. in 2019 and to 0.045 ± 0.019 R.U. in 2022 (Fig. S1d).

**Fig. 4:**
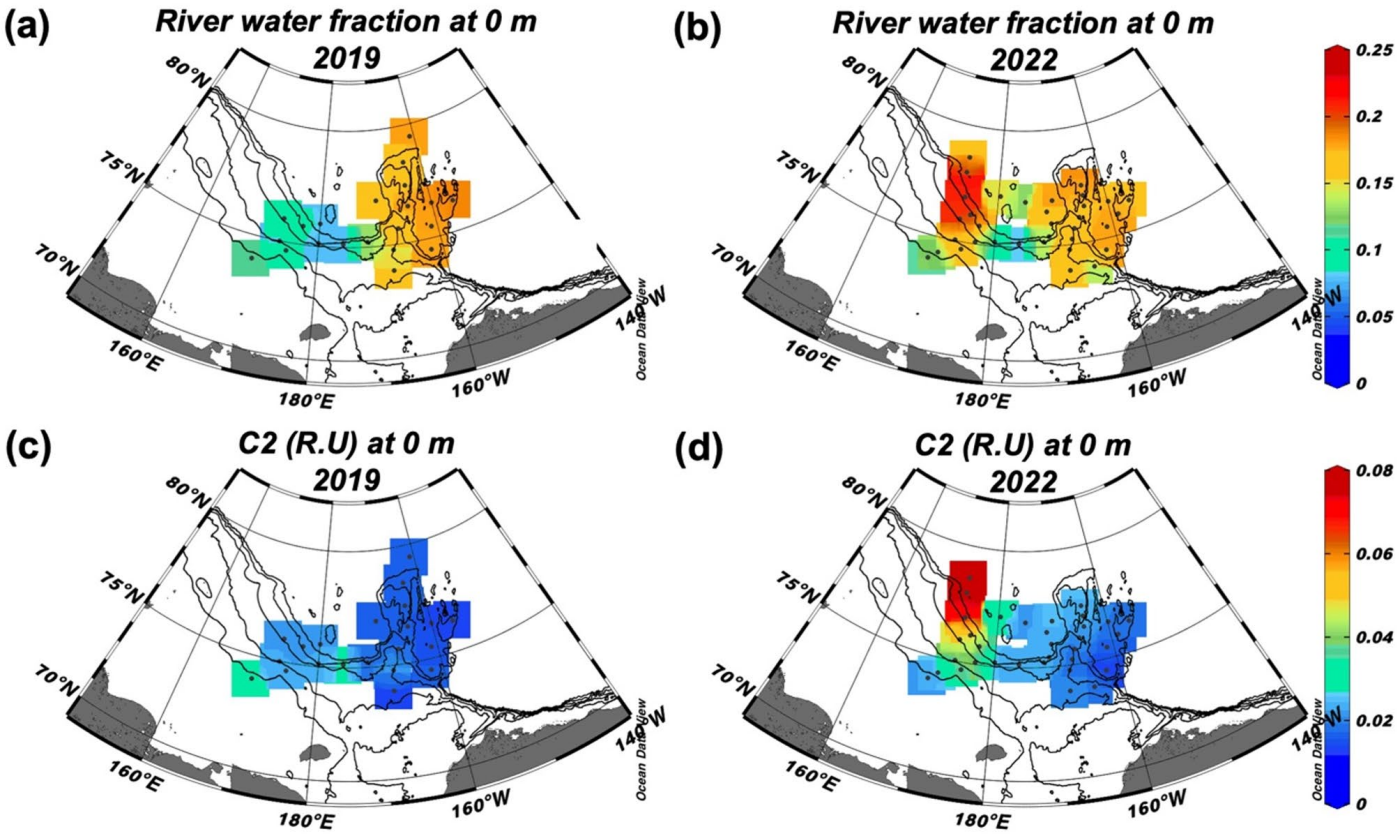
Surface distributions of (**a**,**b**) river water fraction and (**c**,**d**) terrestrial-derived FDOM (C2) (R.U) during the summers of 2019 (left panels) and 2022 (right panels).

**Fig. 5:**
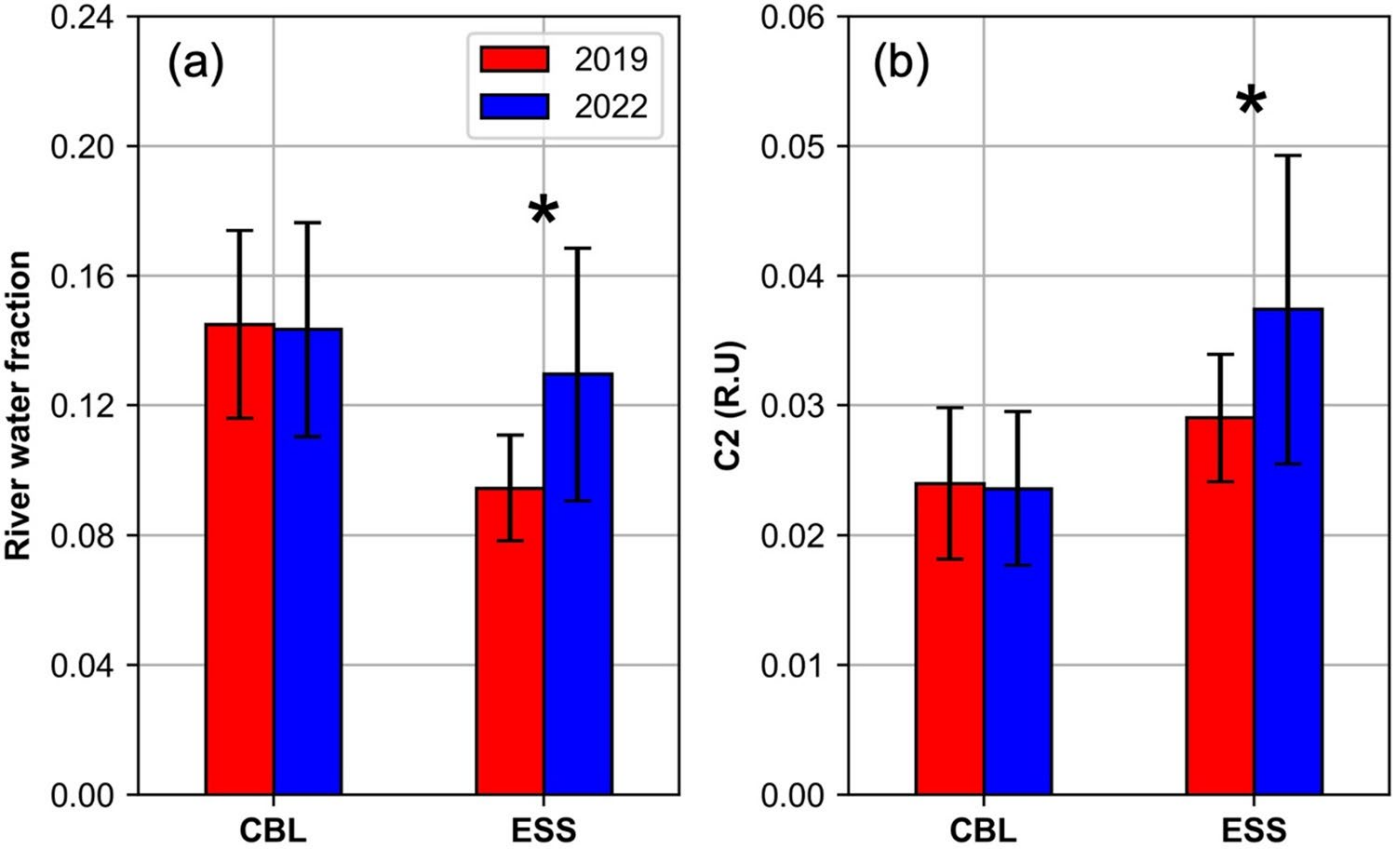
Boxplots of (**a**) river water fraction and (**b**) terrestrial-derived FDOM (C2) (R.U) in the surface waters (< 50 m) of the Chukchi Borderland (CBL) and East Siberian Sea (ESS) at the 18 selected stations (12 in the CBL and 6 in the ESS) where samples were collected in both 2019 (red) and 2022 (blue) (see Fig. 1c for the selected sampling locations). Asterisks above the boxplots indicate statistically significant differences between the two years (*p* < 0.001).

**Fig. 6:**
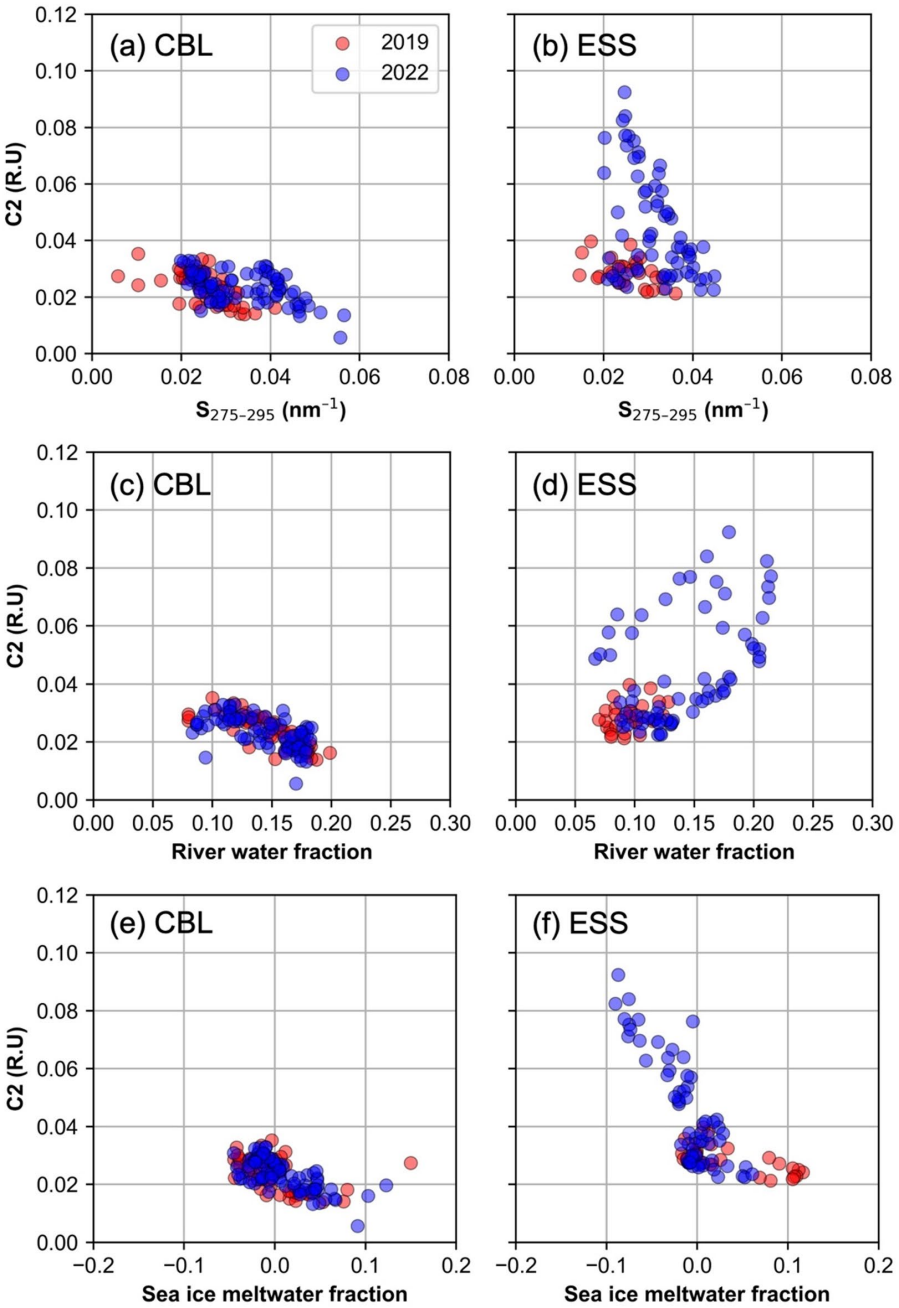
Scatterplots of terrestrial-derived FDOM (C2) (R.U) versus (**a**,**b**) spectral slope of chromophoric DOM between 275 and 295 nm (S_275–295_) (nm^–1^), (**c**,**d**) river water fraction (*f*_river_), and (e, f) sea ice meltwater fraction (*f*_sim_), in the surface waters (< 50 m) of the Chukchi Borderland (CBL) (left panels) and the East Siberian Sea (ESS) during the summers of 2019 (red) and 2022 (blue).

This trend was clearly evident in the surface distribution (Fig. 4). To ensure a robust comparative analysis, we focused on 18 stations (12 in the CBL and 6 in the ESS) sampled at depths within 50 m during both years (Fig. 1c). This analysis confirmed significant increases in both *f*_river_ (37%) and terrestrial humic-like C2 (29%) in ESS surface waters (t-test, *p* < 0.001), while values in CBL surface waters remained stable (*p* = 0.81 for *f*_river_ and *p* = 0.75 for C2) (Fig. 5). The enhanced terrestrial signal in the ESS in 2022 was associated with relatively lower spectral slope coefficients of chromophoric DOM (CDOM) between 275 and 295 nm (S_275-295_ < 0.035 nm^–1^; Fig. 6b), indicating a higher molecular weight CDOM, as lower S_275-295_ values correspond to an increase in its molecular weight^71^. Such low S_275-295_ values, associated with high C2 values, were not detected in the CBL in both 2019 and 2022, as well as in the ESS in 2019 (Fig. 6a,b). Furthermore, a significant positive correlation between *f*_river_ and C2 was observed in ESS in 2022 (*r* = 0.68, *p* < 0.001) (Fig. 6d). This correlation was notably weaker in the ESS in 2019 and in the CBL during both years (Fig. 6c,d). This result suggests that the increase in high-molecular-weight terrestrial humic-like C2 in the ESS in 2022 was closely associated with elevated *f*_river_ values. Moreover, a strong negative correlation between C2 intensities and sea-ice meltwater fraction (*f*_sim_) (*r* = –0.68, *p* < 0.001) in the ESS in 2022 (Fig. 6f), which was not observed in the CBL (Fig. 6e). Considering that *f*_sim_ values below zero indicate brine rejection from sea ice and that this process facilitates the transport of tDOM into the halocline layer^5,65,72^, the strong negative correlation between C2 intensities and *f*_sim_ (Fig. 6f) suggests that terrestrial-derived C2 can be transported into the halocline layer via brine rejection processes.

Given the proximity of the ESS to major Eurasian rivers, namely the Indigirka, Kolyma, and Lena (Fig. 1a), it is important to consider the influence of river discharge variability on the observed increases in *f*_river_ and terrestrial C2. The Lena River is the largest river discharging into the Laptev Sea, with a discharge of ~ 566 km^3^/yr^73^. Its freshwater is transported eastward into the ESS via the Laptev Sea. Meanwhile, the Indigirka and Kolyma rivers, with mean discharges of 55 km^3^/yr and 120 km^3^/yr, respectively^74^, flow directly into the ESS. Collectively, these three rivers serve as the primary freshwater sources for the ESS, significantly contributing to its freshwater budget^41^. To assess this variability between 2019 and 2022, we examined river discharge data from the Arctic Great Rivers Observatory (ArcticGRO)^75^. ArcticGRO data indicate that total river discharge from these three rivers was 372 km^3^ in 2019 and 428 km^3^ in 2022, reflecting a 15% increase over the seven-month period (January–July) preceding our sampling (Fig. S2). Despite this increase, it was still lower than the observed 37% increase in *f*_river_ and 29% increase in C2 within the ESS. Moreover, the difference in river discharge between these two years was not statistically significant (t-test, *p* = 0.62).

### Backward trajectory analysis of ESS surface waters

**Fig. 7:**
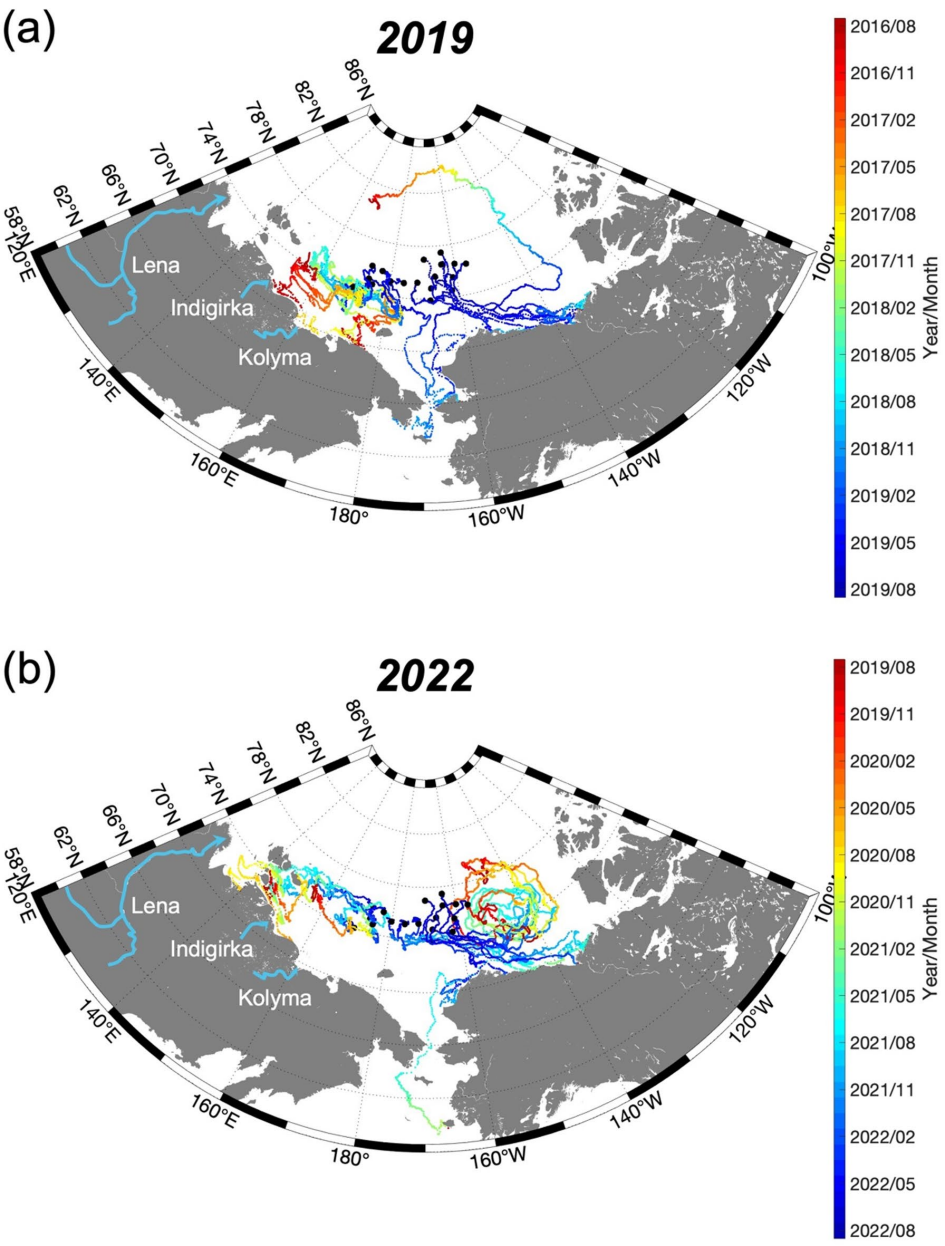
Three-year backward-in-time trajectories of surface water, launched from sampling locations (black dots) in (**a**) 2019 and (**b**) 2022. The colors along the trajectories represent the time in year/month, as indicated by the color bar.

To identify the source of river water and tDOM observed in the eastern ESS during both 2019 and 2022, we conducted backward-in-time trajectory analysis using a Lagrangian particle tracking method (Fig. 7). This analysis revealed fundamentally different source regions and transport pathways for river water and tDOM between the strong and weak BG periods.

During the strong BG period of 2019, eastern ESS surface waters originated predominantly from the western ESS region, influenced primarily by discharge from the Indigirka and Kolyma rivers (Fig. 7a). In contrast, under the weak BG conditions of 2022, backward trajectory analysis indicated that eastern ESS surface waters originated predominantly from the southeastern Laptev Sea, particularly areas near the Lena River delta (Fig. 7b). This shift in source waters from the Indigirka/Kolyma-influenced region to the Lena-influenced region between 2019 and 2022 provides a plausible explanation for the observed 37% increase in *f*_river_ and 29% increase in tDOM. Because the Lena River transports higher concentrations of terrestrial material than the Indigirka and Kolyma rivers, this shift results in greater tDOM input to the eastern ESS, consistent with previous findings^69^.

### Atmospheric drivers of surface water variability in the ESS

**Fig. 8:**
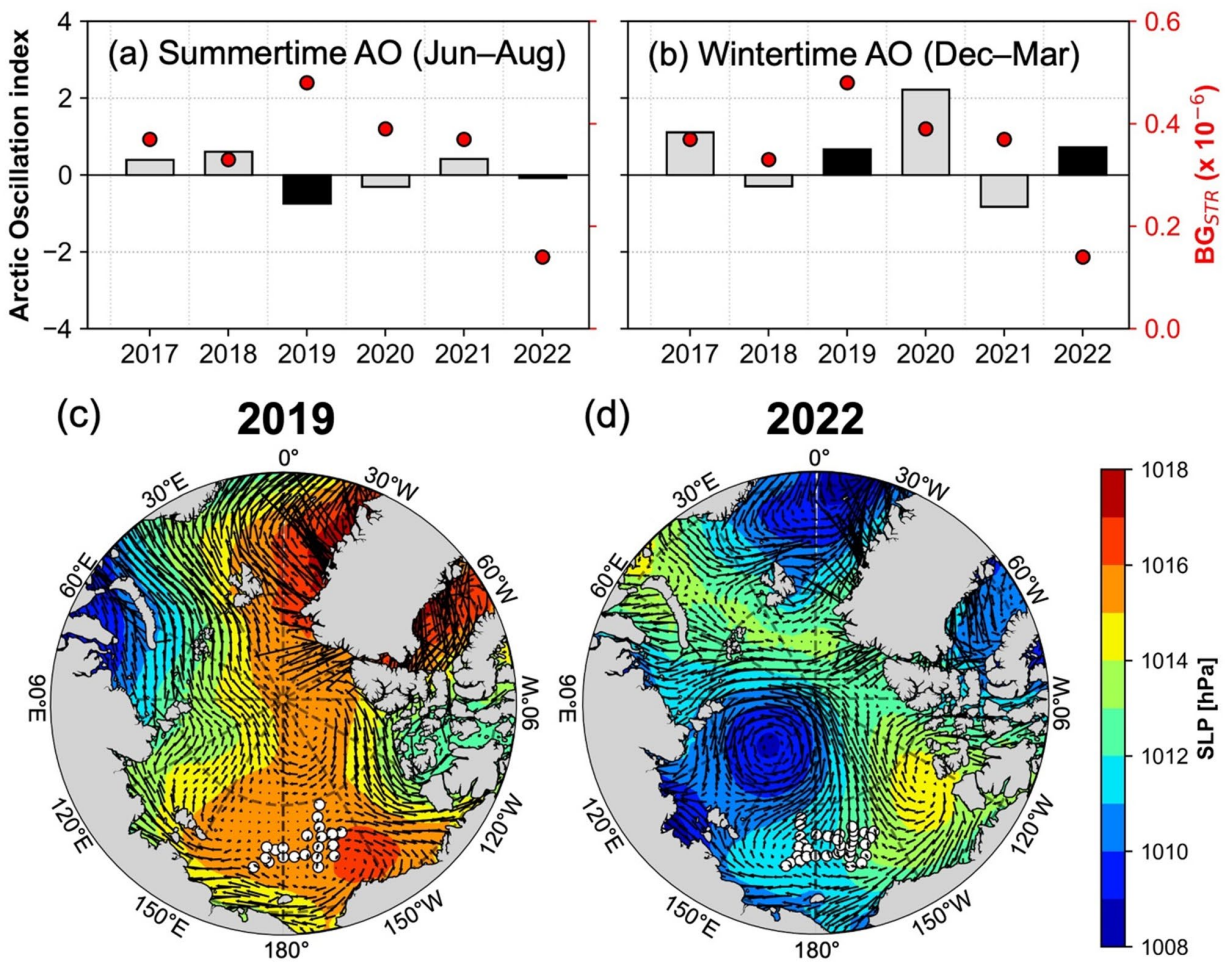
Atmospheric conditions in the Arctic Ocean during 2019 and 2022. Boxplots of the Arctic Oscillation (AO) index averaged over (**a**) the summer months (June–August) and (**b**) the winter months (December–March) for each year from 2017 to 2022. Black bars highlight the averaged AO indices for 2019 and 2022, while red circles represent the Beaufort Gyre strength (BG_STR_). June–August mean fields of 10-m wind vectors (m/s, vectors) and sea level pressure (SLP) (hPa, color-shaded) for (**c**) 2019 and (**d**) 2022. White circles indicate the sampling stations for each year.

The AO is a recognized driver of Arctic Ocean circulation^29^, and variations in both its summertime and wintertime indices have been linked to changes in river water distribution^26,36,76–80^. Given its influence on river water distribution, we investigated AO variability during both seasons. Summertime AO indices revealed a pronounced negative AO (–0.738) in 2019 and a near-neutral AO (–0.073) in 2022 (Fig. 8a). However, under negative or near neutral AO conditions, the TPD typically maintains its position over the Lomonosov Ridge^4^, limiting the eastward transport of Lena River water into the eastern ESS. We also examined the wintertime AO. Surprisingly, wintertime AO indices showed no significant difference between 2019 (0.67) and 2022 (0.72; *p* = 0.94) (Fig. 8b). These findings suggest that AO variability during both summer and winter is insufficient to explain the observed changes in river water and tDOM distribution.

Given the limited influence of the AO, we next investigated regional atmospheric patterns as a potential driver of the observed changes in river water and tDOM distribution. Analysis of these patterns revealed distinct differences in surface pressure fields and wind forcing between the two periods (Fig. 8c,d). The summer of 2019 was characterized by a strong dipole pattern, with low SLP over the Kara Sea coupled with high SLP over the Beaufort Sea and Greenland. This pressure configuration generated winds that transported surface waters from the Laptev Sea toward Fram Strait, effectively limiting the eastward expansion of Lena River water into the eastern ESS (Fig. 8c). In contrast, the atmospheric pattern shifted markedly in 2022, with weakened pressure gradients across the Arctic basin (Fig. 8d). Notably, enhanced cyclonic circulation over the Eurasian Basin extended toward the Beaufort Sea, disrupting the 2019 dipole pattern. This circulation shift, combined with predominant southwesterly winds over the ESS, generated southeasterly Ekman transport. This new circulation regime provided a direct physical mechanism for the observed expansion of Lena River-influenced water into the eastern ESS, explaining both the increased *f*_river_ and elevated tDOM values.

## Discussion

Our analysis reveals that *f*_river_ and terrestrial humic-like C2 in the eastern ESS increased by 37% and 29% in the ESS, respectively, between 2019 and 2022. Given that river discharge from adjacent rivers did not differ significantly between these two years (t-test, *p* = 0.62), riverine input alone cannot fully account for these changes. A comparison with previous studies^5,42^, provides further evidence supporting this interpretation. In 2022, *f*_river_ values in the eastern ESS were anomalously high (mean: 0.170 ± 0.033), a value not observed in previous years: 2017 (mean: 0.118 ± 0.019)^5^, 2018 (mean: 0.108 ± 0.034)^42^, and 2019 (mean: 0.092 ± 0.014). Notably, total river discharge from the three adjacent rivers during January–July was highest in 2017 (487 km^3^), followed by 2018 (459 km^3^), 2022 (428 km^3^), and 2019 (372 km^3^) (Fig. S2). However, despite the highest river discharge occurring in 2017, *f*_river_ values in the eastern ESS were highest in 2022, suggesting that additional mechanisms beyond riverine input are responsible for these changes.

A previous study^37^ investigated tDOM distribution in the Siberian shelf seas during the summer of 2019, particularly focusing on the interaction between atmospheric forcing and tDOM. Their findings indicated that southeasterly winds over the Laptev Sea in 2019 blocked Lena River-influenced surface waters from extending into the western ESS, while anomalously low river discharge into the Laptev Sea and ESS further contributed to reduced tDOM in the region. These findings are consistent with our observations from 2019. However, our study expands on this by examining how changes in atmospheric forcing between 2019 and 2022 influenced the distribution of river water and tDOM in the eastern ESS.

Traditionally, large-scale drivers such as the AO have been emphasized as key regulators of river water distribution^26,77,81^. While recent studies highlight the role of local wind fields in Arctic Ocean dynamics^82–84^, the AO remains a key driver, as other recent studies have demonstrated its continued influence on Arctic Ocean dynamics^30,79,80,85,86^. We therefore examined the influence of AO index on the distribution of river water and tDOM and found negligible AO variability between 2019 and 2022 (Fig. 8a,b). This suggests that neither AO nor river discharge was a major driver in regulating the distribution of river water and tDOM in the eastern ESS. A comparison between the 2017 study^5^ and the 2022 results reinforces our findings, showing that *f*_river_ values in the eastern ESS were higher in 2022 (Figs. 8a, b, and S2) despite higher AO and river discharge in 2017.

Given that these traditional drivers could not fully explain our observations, we conducted a three-year backward trajectory analysis to identify specific source regions and transport pathways. In 2019, surface waters in the eastern ESS primarily originated from the Indigirka and Kolyma rivers, whereas in 2022, they were predominantly influenced by the Lena River (Fig. 7). To further evaluate how short-term BG variability on a one-year timescale impacts the lateral advection of river water and tDOM, we analyzed one-year backward trajectories (Fig. 7, blue scale bar). Even on this shorter timescale, notable differences emerged. In 2019, surface waters were located between approximately 160 and 180°E one year prior, whereas in 2022, they were positioned farther west near 150°E. These shifts suggest that intra-annual atmospheric circulation changes played a crucial role in modulating the distribution of river water and tDOM in the eastern ESS.

The contrasting water source regions identified in our backward trajectory analysis can be explained by distinct atmospheric circulation patterns between 2019 and 2022, particularly the Arctic Dipole (AD) pattern. The AD is a pressure pattern characterized by one pole situated between the Kara and Laptev seas and the other over the Canadian Archipelago^87^. In 2019, a positive AD phase was observed, characterized by low pressure over the Kara Sea and high pressure over the Beaufort Sea^87–89^ (Fig. 8c). During this phase, the TPD strengthened^87–89^ and directed Lena River water toward the Lomonosov Ridge^36,89^. This circulation pattern limited the ESS to receiving inputs primarily from the Indigirka and Kolyma rivers, which have lower discharge and approximately twofold lower DOM concentrations than the Lena River^69,90^. Consequently, the resulting DOM distribution reflected the influence of these rivers with lower tDOM sources. The 2019 positive AD also contributed to anomalously low sea ice concentrations (Fig. S3) through two mechanisms: increased oceanic heat flux through the Bering Strait and strong easterly winds that transported sea ice from the western to the eastern Arctic Ocean^88^. These winds were so significant that they led to the second-lowest sea ice extent recorded during the satellite era^88^. The combined effects of the positive AD—reduced sea ice cover and enhanced eastward transport via the TPD—facilitated the transport of surface waters from the Indigirka and Kolyma rivers into the ESS.

In contrast, strengthened cyclonic circulation over the Eurasian Basin in 2022 fundamentally altered the transport pathways of river water and tDOM. As the BG weakened, eastward transport of water masses from the Laptev Sea into the eastern ESS increased, as confirmed by our backward trajectory analysis (Fig. 7b). This shift in circulation significantly affected tDOM distribution. The Lena River, which dominates freshwater input to the Laptev Sea, has extensive permafrost coverage, high tDOM concentrations, and limited microbial degradation within its watershed^11,69^, contributing to elevated values of high-molecular-weight CDOM^78^. The redirection of surface water origins toward the Laptev Sea in 2022 thus increased high-molecular-weight CDOM in the eastern ESS (Figs. 2h and 4d). This circulation-driven shift is further supported by changes in water mass distribution, with a weakened intrusion of Pacific-origin water and an enhanced intrusion of Atlantic-origin water into the ESS in 2022 compared to 2019 (Fig. 2b,d, f). Previous studies have suggested that enhanced cyclonic circulation can contribute to the retreat of Pacific-origin water and expansion of Atlantic-origin water^27,91,92^. More recently, strengthened cyclonic circulation over the Eurasian Basin was found to drive an eastward extension of Atlantic-origin lower halocline water into the ESS^32^. Thus, the shrinking BG facilitated multiple related changes: the retreat of Pacific-origin water, the expansion of Atlantic-origin water into the ESS, and the eastward transport of Lena River water with its high tDOM load.

The decline in Arctic sea ice has the potential to increase BG variability^43^, which in turn influences shifts in tDOM advection, as demonstrated in this study. If this BG weakening persists, the high-molecular-weight terrestrial CDOM from the Lena River could increasingly enter the BG region, subsequently affecting biogeochemical processes. One plausible implication is the alteration of DOM composition, as the BG’s long residence time (approximately 20 years)^93,94^ would subject this material to extended photodegradation^95,96^, potentially accelerating its transformation into lower-molecular-weight compounds^97,98^. Additionally, this transformation process could result in CO_2_ outgassing^99,100^, contributing to the carbon cycle. Beyond surface water processes, our observation of the significant negative correlation between terrestrial-derived C2 and *f*_sim_ (Fig. 6f) suggests the downward export of tDOM into the halocline layer. This process could facilitate the sequestration of tDOM into the deeper Arctic Ocean, where it can be incorporated into large-scale circulation patterns, including the Atlantic Meridional Overturning Circulation (AMOC)^101^. While Siberian Shelf waters currently contribute only about 1% to Denmark Strait Overflow Water^101^, shifts in Arctic circulation patterns could alter this contribution. Given the AMOC’s crucial role in global carbon redistribution^102^, changes in the transport and modification of tDOM in the Arctic could have far-reaching effects on carbon cycling at both regional and global scales^10,93^.

## Methods

### Field sampling and hydrographic measurements

We conducted hydrographic surveys in the WAO during two contrasting periods of BG_STR_: August 9–24, 2019, and July 27–August 19, 2022, aboard the Korean icebreaker IBR/V Araon. The study region encompassed two distinct areas: the ESS and CBL, with 21 stations sampled in 2019 and 34 in 2022. At each station, we obtained vertical profiles of temperature and salinity using a SeaBird Electronics SBE911 + conductivity-temperature-depth (CTD) system. Salinity from sensor (practical salinity unit; S_P_) was converted to absolute salinity (S_A_) using the equation proposed by Millero^103^: S_A_ (g/kg) = 35.165/35.000 × S_P_.

Seawater samples were collected using a rosette system equipped with 10 L Niskin bottles. For nutrient analysis, unfiltered samples were directly collected into pre-washed 50 mL conical tubes, stored at 4 °C in the dark, and analyzed within three days. DOM and oxygen isotope (*δ*^18^O) samples were gravity-filtered through pre-combusted (550 °C, 6 h) GF/F filters using acid-cleaned equipment. DOM samples were sealed in pre-combusted glass ampoules and stored at –24 °C in darkness, while *δ*^18^O samples were stored at 4 °C in acid-cleaned vials until analysis.

### Chemical and isotopic analyses

Nutrient concentrations (NO_2_^−^ + NO_3_^−^ and PO_4_^3−^) were measured using a QuAAtro auto-analyzer following the Joint Global Ocean Flux Study (JGOFS)^104^ protocols, with accuracy verified using reference material for nutrients in seawater (Lot No. “BV”, KANSO Technos Co., Ltd., Osaka, Japan). The accuracy of the measured NO_2_^−^ + NO_3_^−^ and PO_4_^3−^ concentrations, expressed as relative standard deviation, were ± 1.0% at 35.33 μmol/kg and ± 0.89% at 2.514 μmol/kg, respectively. The detection limits, calculated as three times the standard error of the intercept-to-slope ratio of the calibration line, were 0.27 and 0.21 μmol/kg for NO₂⁻ + NO₃⁻ and PO₄^3−^, respectively^95^. We calculated N^*^, a denitrification tracer, according to the following equation^105^:

$${\text{N}}^{*} = \left[ {\left( {{\text{NO}}_{2}^{ - } + {\text{NO}}_{3}^{ - } } \right){-}16{\text{PO}}_{4}^{3 - } + 2.9} \right] \times 0.87\,\upmu {\text{mol}}/{\text{kg}}$$

*δ*^18^O were determined using CO₂ equilibration and mass spectrometry (Isoprime) at Korea Basic Science Institute, achieving precision better than 0.1‰. The *δ*^18^O values were calculated as:

$$\delta^{18} {\text{O}} = \left[ {\left( {^{18} {\text{O}}/^{16} {\text{O}}_{{{\text{sample}}}} /^{18} {\text{O}}/^{16} {\text{O}}_{{\text{V - SMOW}}} } \right){-}1} \right] \times 1000$$

We used *δ*^18^O and salinity data to calculate freshwater fractions through the following mass balance equations^106^:

1 $$f_{{{\text{sw}}}} + f_{{{\text{sim}}}} + f_{{{\text{river}}}} = 1$$

2 $$f_{{{\text{sw}}}} \delta^{18} O_{{{\text{sw}}}} + f_{{{\text{sim}}}} \delta^{18} O_{{{\text{sim}}}} + f_{{{\text{river}}}} \delta^{18} O_{{{\text{river}}}} = \delta^{18} O_{{{\text{obs}}}}$$

3 $$f_{{{\text{sw}}}} \cdot S_{{{\text{sw}}}} + f_{{{\text{sim}}}} \cdot S_{{{\text{sim}}}} + f_{{{\text{river}}}} \cdot S_{{{\text{river}}}} = S_{{{\text{obs}}}}$$

where *f* and *S* represent the fraction and salinity of each component (seawater, sea-ice melt, and river water), respectively. The subscript ‘obs’ denotes observed values. We used established Arctic Ocean end-member values: *S* = 34.8, 4, and 0 psu; *δ*^18^O = 0.28, − 2, and − 20‰ for seawater, sea-ice melt, and river water, respectively.

### Optical characterization of DOM

DOM optical properties were characterized using both absorption and fluorescence spectroscopy. Absorption spectra (200–800 nm) were measured using a Shimadzu UV-2600 spectrophotometer, with daily Milli-Q water blanks. The absorption coefficient was calculated as follows^107^:

4 $${\text{a}}\left( \lambda \right) = 2.303A\left( \lambda \right)/{\text{L}}$$

where a is the absorption coefficient (m^−1^), A is the absorbance, and L is the path length (m). The spectral slope coefficient (S_275–295_) was determined using^71^:

5 $${\text{a}}\left(\uplambda \right) = {\text{a}}\left( {\uplambda _{0} } \right)\exp \left[ {{-}S\left( {\uplambda {-}\uplambda _{0} } \right)} \right]$$

where λ₀ is the reference wavelength. S_275–295_ serves as a reliable optical proxy for the average molecular weight of CDOM^71,108,109^ due to its well-established negative correlation with molecular weight of DOM^71^.

Fluorescence measurements employed a Hitachi F-7100 spectrofluorometer with a 1 cm quartz cuvette. Ex wavelengths were scanned from 250 to 500 nm in 5 nm increments, while Em wavelengths were scanned from 280 to 550 nm in 1 nm increments. Both Ex and Em slit widths were fixed at 5 nm. The EEM of Milli-Q water was subtracted from each sample’s EEMs. Fluorescence intensities were corrected based on the area under the Raman peak of Milli-Q water (Ex = 350 nm), which was measured daily, and normalized to Raman Unit (R.U.)^110^. PARAFAC was conducted in MATLAB R2024b (MathWorks) using the drEEM toolbox^111^. Raman and Rayleigh scatter peaks were removed using the *smootheem* function. The model was constrained to nonnegative values with a convergence criterion of 1 × e^–6^ and tested for 3 to 7 components. The number of fluorescent components was determined based on split-half analysis.

### River discharge data

We utilized river discharge data from 2017 to 2022 provided by Arctic Great Rivers Observatory (ArcticGRO) Discharge Dataset (https://arcticgreatrivers.org/discharge/)^75^. Our analysis focused on the three rivers influencing the ESS: the Indigirka, Kolyma, and Lena rivers. For each year, the total combined discharge was calculated as the sum of the January–July average discharge for these three rivers.

### Circulation analysis and atmospheric data

Surface water trajectories were reconstructed using a backward-in-time Lagrangian particle tracking method, following the approach of Kim et al.^112^. While Kim et al.^112^ utilized ice motion vectors to track sea ice movement, our study applied a similar methodology to surface water tracking, replacing ice motion vectors with velocity fields from the Global Ocean Reanalysis (GLORYS 12) (product id: GLOBAL_MULTIYEAR_PHY_001_030), obtained from the Copernicus Marine Environment Monitoring Service (https://marine.copernicus.eu/). Atmospheric conditions were analyzed using NOAA’s AO index and NASA Modern-Era Retrospective Analysis for Research and Applications, version 2 (MERRA-2) reanalysis data for SLP and wind fields. We extracted DOT for the calculation of BG_STR_ from 2017 to 2022 using Arctic Ocean Physics Reanalysis (product id: ARCTIC_MULTIYEAR_PHY_002_003), which has a spatial resolution of 3 × 3 km and a daily temporal resolution. The dataset is available from the Copernicus Marine Environment Monitoring Service (https://marine.copernicus.eu/). DOT values for each year were averaged over the respective sampling periods (August 10–24, 2017; August 6–24, 2018; August 9–25, 2019; August 6–27, 2020; July 24–August 16, 2021; and July 27–August 19, 2022). The BG extent (BG_EXT_) was defined as the largest closed DOT contour surrounding the maximum DOT (DOT_max_)^44^. The BG_STR_ was calculated as the difference between the DOT_max_ and minimum DOT (DOT_min_), divided by the mean radius of the BG^44^.

### Statistical analysis

All statistical analyses were performed using Python (version 3.9.19) with the SciPy library (version 1.13.1). The normality of data distributions was assessed using the Kolmogorov–Smirnov test (two-tailed, α = 0.05). When normality was confirmed, independent t-tests were conducted to compare means between groups.

## Electronic supplementary material

Below is the link to the electronic supplementary material.

Supplementary Material 1

## Data Availability

All data are available on the KOPRI data servers accessible through 10.22663/KOPRI-KPDC-00002763.
