# Supplementary material for: Interannual variability in terrestrial dissolved organic matter advection to the eastern East Siberian Sea under contrasting Beaufort Gyre conditions
Source: Sci Rep. 2025 Jul 2;15:23084. doi: 10.1038/s41598-025-07732-w (PMC12219028; doi:10.1038/s41598-025-07732-w)
Supplement: Supplementary file 1 — Supplementary Material 1 [file 41598_2025_7732_MOESM1_ESM.docx]

*Scientific Reports*

Supporting Information for

**Interannual variability in terrestrial dissolved organic matter advection to the eastern East Siberian Sea under contrasting Beaufort Gyre conditions**

Mi Hae Jeon^1^, Jinyoung Jung^1,*^, Juyoung Son^1,2^, Kyoung-Ho Cho^1^ & Eun Jin Yang^1^

^1^Korea Polar Research Institute, 26 Songdomirae‑ro, Yeonsu‑gu, Incheon 21990, Republic of Korea

^2^Division of Earth and Environmental System Science, Pukyong National University, 45 Yongso-ro, Nam-gu, Busan 48513, Republic of Korea

*Correspondence: Jinyoung Jung ([jinyoungjung@kopri.re.kr](mailto:jinyoungjung@kopri.re.kr))

**Contents of this file**

Figures S1 to S3

This Supporting Information includes three supplementary figures referenced in the main text of the article.


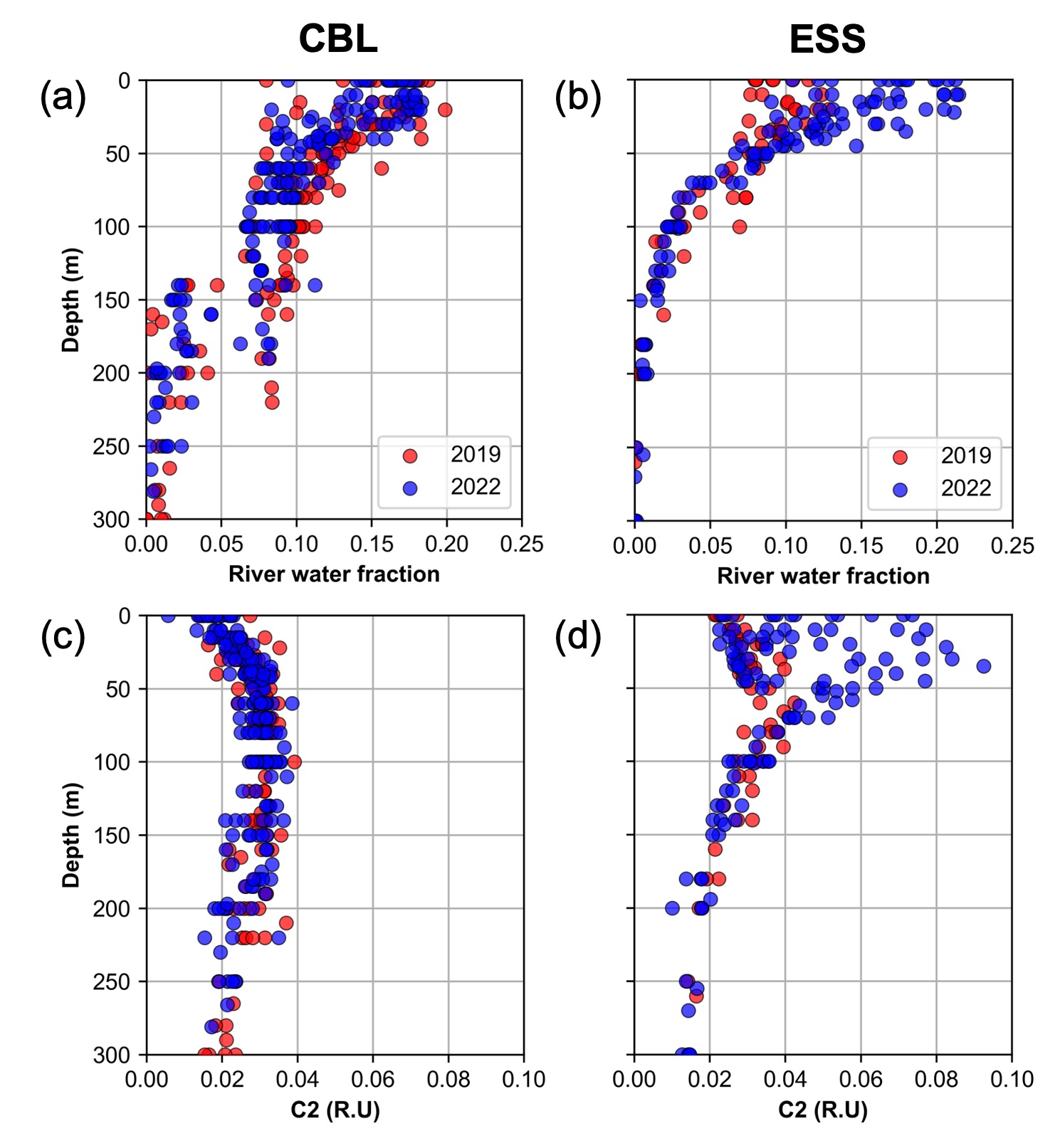


Figure S1. Vertical profiles of (a, b) river water fraction and (c, d) terrestrial-derived FDOM (C2) (R.U) observed in the Chukchi Borderland (CBL, left panels) and the East Siberian Sea (ESS, right panels) during the summers of 2019 (red circles) and 2022 (blue circles).


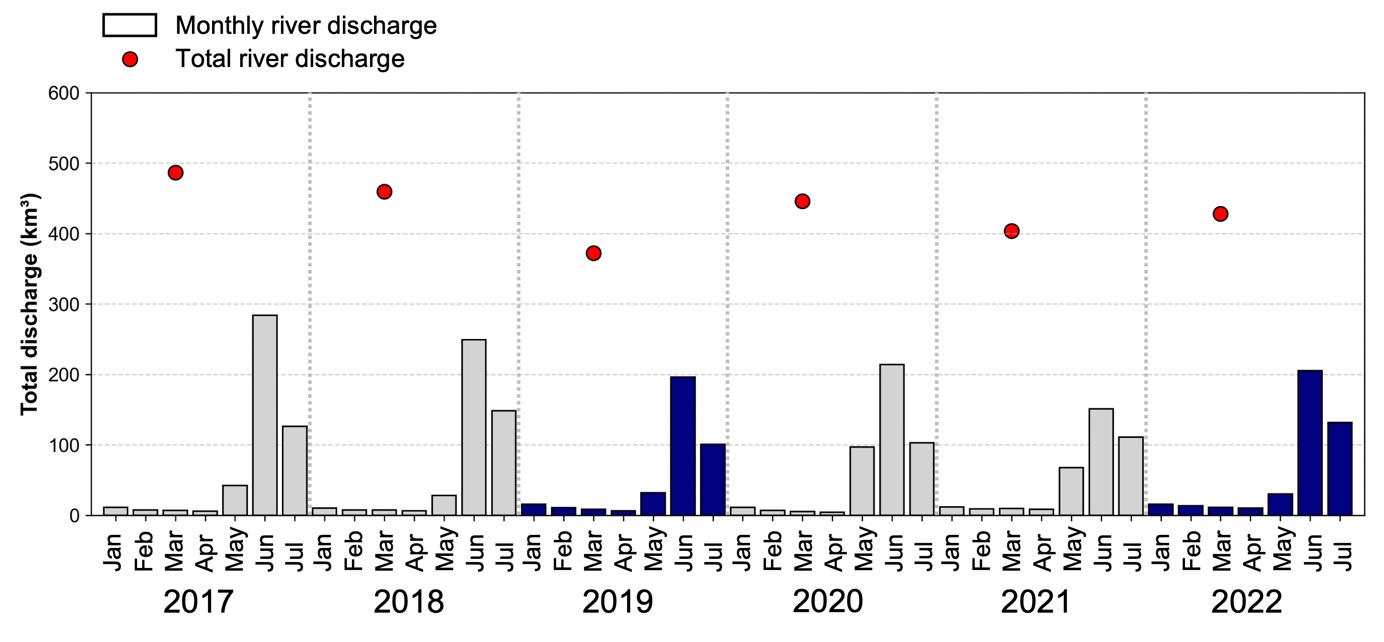


Figure S2. Monthly river discharge from January to July for each year from 2017 to 2022. The bars represent the combined monthly discharge values (sum of the monthly averages from the Indigirka, Kolyma, and Lena rivers). Dark blue bars highlight the values for 2019 and 2022. The red dots indicate the total combined discharge (sum of the January-July average discharge for each of the three rivers) for each respective year.


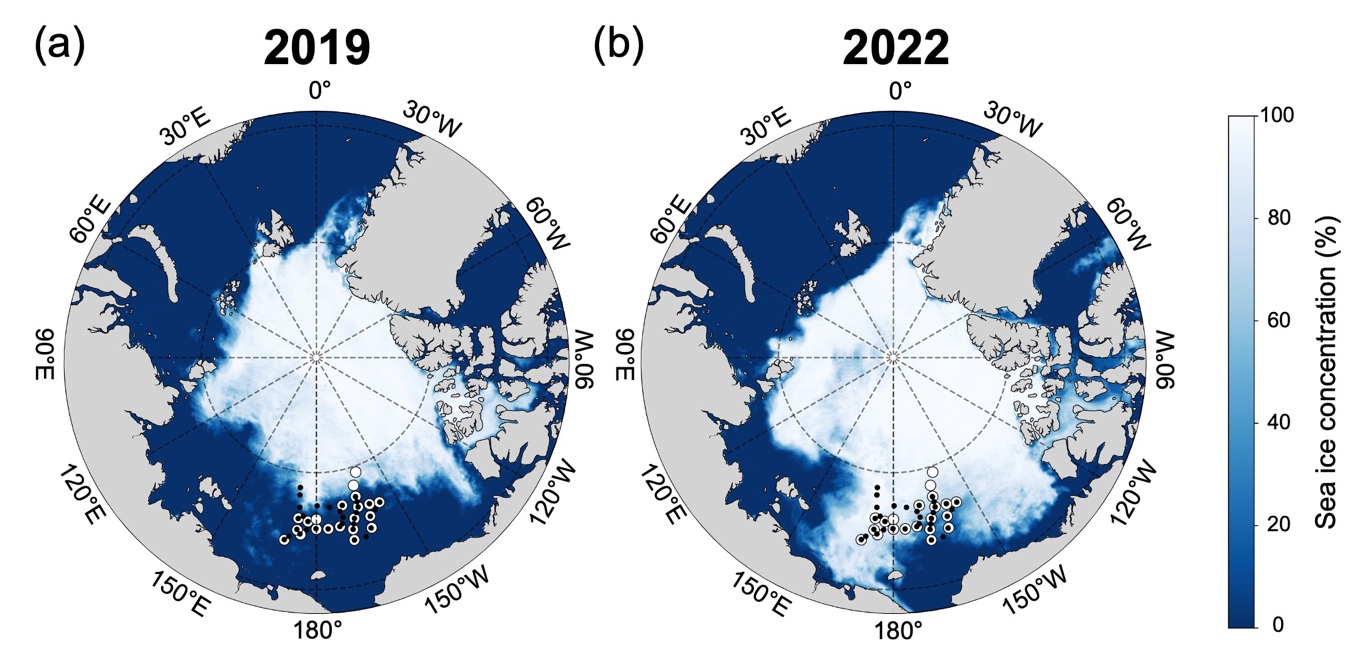


Figure S3. Averaged sea ice concentrations in the Arctic Ocean during the sampling periods in (a) 2019 and (b) 2022. The locations of sampling stations are superimposed onto sea ice concentration data derived from Advanced Microwave Scanning Radiometer (AMSR) 2. White and black circles represent seawater sampling stations in the summers of 2019 and 2022, respectively.
